# Altered levels of circulating miRNAs are associated *Schistosoma japonicum* infection in mice

**DOI:** 10.1186/s13071-015-0806-5

**Published:** 2015-04-01

**Authors:** Lihui Zhu, Jinwei Dao, Xiaoli Du, Hao Li, Ke Lu, Jinming Liu, Guofeng Cheng

**Affiliations:** Shanghai Veterinary Research Institute, Chinese Academy of Agricultural Sciences; Key Laboratory of Animal Parasitology, Ministry of Agriculture, 518 Ziyue Road, Shanghai, China

**Keywords:** Schistosome, MicroRNAs, Host-parasite interaction, Pathogenesis, Schistosomiasis

## Abstract

**Background:**

Dioecious flatworms of the genus *Schistosoma* causes schistosomiasis, which is a major public health problem in developing countries. Acquiring detailed knowledge of schistosome-host interactions may aid in the development of novel strategies for schistosomiasis control. MicroRNAs (miRNAs) are involved in processes such as development, cell proliferation, metabolism, and signal transduction. Circulating miRNAs not only serve as a novel class of biomarkers of many diseases but also regulate target gene expression in recipient cells, which are similar to hormones.

**Methods:**

In the present study, we used miRNA microarrays to determine the profile of circulating miRNAs associated with *S. japonicum* infection of mice. The biological functions of the altered levels of miRNAs and their target genes were predicted using bioinformatics. Expression levels of selected miRNAs and their target genes were further analyzed by quantitative RT-PCR.

**Results:**

Our study identified 294 and 189 miRNAs in infected mice that were expressed in two independent experiments at levels ≥ 2-fold higher or ≤ 0.5-fold lower, respectively, compared with uninfected mice. Thirty-six of the same miRNAs were detected in these analyses. Moreover, pathway analyses indicated that most of these miRNAs are putatively involved in signaling pathways associated with pathogenesis, such as Wnt and MAPK signaling. Further, we show an inverse correlation between the circulating levels of these miRNAs and their target genes, suggesting that changes in miRNA expression may cause aberrant expression of genes such as Creb1 and Caspase-3 in mice infected with *S. japonicum*.

**Conclusions:**

Our study shows significant differences in the levels of circulating miRNAs between *S. japonicum* infected mice and uninfected mice. In particular, the altered levels of miR-706 and miR-134-5p were associated with altered levels of expression of the Caspase-3 and Creb1 genes, respectively, suggesting that circulating miRNAs may serve as important mediators of the pathology of hepatic schistosomiasis. Additionally, our results are expected to provide new insights for further understanding the mechanisms of schistosome-host interaction that may facilitate in the development of novel interventions for alleviating the symptom of *S. japonicum* infection as well as for preventing and treating schistosomiasis.

**Electronic supplementary material:**

The online version of this article (doi:10.1186/s13071-015-0806-5) contains supplementary material, which is available to authorized users.

## Background

Millions of people worldwide suffer from schistosomiasis particularly those residing in developing countries [1,2], and this disease is one of the most important public health concerns in China [3]. However, no vaccine is available for preventing schistosomiasis. Treatment of schistosomiasis and controlling morbidity relies mainly on a single drug, praziquantel, which provides only partial protection and is ineffective against the young developing stages of schistosomes [4]. Moreover, praziquantel does not prevent reinfection, and there are concerns that large-scale and repeated use of a single drug might select for drug resistant parasites [5,6]. Considering the high incidence and complex biology of schistosomiasis, it is important to define in detail the host–parasite interaction, which should provide valuable clues for developing novel strategies for controlling this disease.

MicroRNAs (miRNAs) are small regulatory RNAs that are involved in numerous biological processes [7]. Evidence suggests that schistosome miRNAs play a potentially regulatory role in worm development and parasitism as well as in drug resistance [8-10]. Circulating miRNAs are similar to hormones, because they serve as biomarkers of the pathogenesis of many diseases, regulate gene expression [11] and may serve as targets of therapy [12-14]. Moreover, adult schistosomes live for long periods in the circulation of their final hosts. Recent studies further demonstrated that pathogen specific miRNAs can be detected in circulation of a final host infected with *S. japonicum* [15-17], suggesting that circulating miRNAs may not only act as important mediators of host-parasite interaction but also serve as a novel class of biomarkers for schistosomiasis diagnosis [18]. In addition, aberrant host miRNAs have also showed to be associated with schistosome infection, implying host miRNAs may be potential indicators of schistosomiasis. For instance, He and co-workers demonstrated that miR-223 was significantly up-regulated in the serum of mice infected with *S. japonicum* and returned to near normal levels on praziquantel treatment [17], implying that up-regulated murine miR-223 may be a biomarker for *Schistosoma* infection. However, knowledge of miRNA functions of host-schistosome interactions and worm parasitism is limited. Consequently, we used miRNA microarrays to determine the profile of circulating miRNAs associated with *S. japonicum* infection at 25 days of post infection (dpi). We expect that the findings will provide valuable clues for a better understanding of the mechanisms of host–schistosome interactions and the identification of alternative biomarkers and drugs targets.

## Methods

### Ethics statement

All animal care and experimental procedures were carried out in strict accordance with the protocol approved by the Ethics and Animal Welfare Committee of the Shanghai Veterinary Research Institute, Chinese Academy of Agricultural Sciences.

### Animals and parasites

BALB/c mice were percutaneously infected with approximately 120 *S. japonicum* cercariae (Anhui isolate, China). Blood samples (approximately 600 μL) from each mouse were obtained from the orbital sinus at 25 dpi. There were 2 groups (uninfected and 25 dpi) with 4 mice in each group. Pools of plasma from 4 *S. japonicum* infected mice and 4 uninfected controls were subjected to miRNA profile analysis.

### Isolation of total RNA

Total RNAs was isolated using TRIzol (Invitrogen) combined with a miRNeasy mini kit (QIAGEN) according to the manufacturer’s instructions. RNA quality and quantity was measured using a NanoDrop 1000 spectrophotometer (Thermo Fisher Scientific). The integrity of the RNA was evaluated using gel electrophoresis, and only RNA preparations with a ratio of absorbance at 260 nm to 280 nm > 1.8 were used.

### MiRNA microarray analyses

RNA samples were labeled using the miRCURY Hy3/Hy5 Power labeling kit (Exiqon, Vedbaek, Denmark) and hybridized to the Exiqon miRCURY LNA Array (v.18.0), which contains 3100 capture probes representing all human, mouse, and rat microRNAs sequences annotated in miRBase 18.0 as well as all related viral microRNAs. Expression data were extracted from the scanned images using GenePix Pro 6.0 software (Axon). Data for replicated miRNAs were averaged, and miRNAs with intensities of ≥ 30 in all samples were chosen for calculating the normalization factor. The data were normalized according to the median normalization value. The microarray experiments were performed by Kangchen Bio-tech, Shanghai, China.

### Statistical analysis of altered levels of miRNAs

The miRNAs that were expressed at ≥ 2-fold higher or ≤ 0.5-fold lower levels in the plasma of infected mice compared with uninfected mice were selected for further analysis. Full details of the miRNA microarray analyses were deposited in the Gene Expression Omnibus (GEO; http://www.ncbi.nlm.nih.gov/geo/) public database with the associated platform accession number GPL16016. The entire microarray data set was MIAME compliant. The raw data are available through GEO under Accession: GSE63135.

### Validation of microarray data using quantitative RT-PCR (qRT-PCR)

Eight altered levels of miRNAs (let-7b-3p, miR-1194, miR-134-5p, miR-1981-3p, miR-210-5p, miR-542-3p, miR-706, and miR-92a-2-5p) were selected for qRT-PCR analysis. Blood samples taken 25 dpi from the infected and uninfected mice were collected from the orbital sinus as described previously [15]. The plasma RNA of pooled blood samples (at least 4 mice) was extracted using a mirVana PARIS Kit (Invitrogen) according to the manufacturer’s instructions. For qRT-PCR analysis, a stem-loop RT primer was used to reverse-transcribe mature miRNAs (Table 1).

**Table 1:** **Primers used for qRT-PCR analysis of miRNA expression**

**MicroRNAs**	**Primer sequences 5'-3'**	**Annealing temperature (°C)**
**Reverse transcription**		
let-7b-3p	GTCGTATCCAGTGCAGGGTCCGAGGTATTCGCACTGGATACGACGGGAAG	
miR-1194	GTCGTATCCAGTGCAGGGTCCGAGGTATTCGCACTGGATACGACAGGATC	
miR-134-5p	GTCGTATCCAGTGCAGGGTCCGAGGTATTCGCACTGGATACGACCCCCTC	
miR-1981-3p	GTCGTATCCAGTGCAGGGTCCGAGGTATTCGCACTGGATACGACGTCAAA	
miR-210-5p	GTCGTATCCAGTGCAGGGTCCGAGGTATTCGCACTGGATACGACCAGTGT	
miR-92a-2-5p	GTCGTATCCAGTGCAGGGTCCGAGGTATTCGCACTGGATACGACGTAATG	
miR-542-3p	GTCGTATCCAGTGCAGGGTCCGAGGTATTCGCACTGGATACGACTTTCGT	
miR-706	GTCGTATCCAGTGCAGGGTCCGAGGTATTCGCACTGGATACGACTTTTTT	
**Quantitative RT-PCR**		
let-7b-3p	F: ATCGTACGTGGGCTATACAAC	61.5
miR-1194	F: ATCGTACGTGGGGAATGAGTA	57
miR-134-5p	F: ATCGTACGTGGGTGTGACTGG	61.5
miR-1981-3p	F: ATCGTACGTGGGCATCTAACC	58
miR-210-5p	F: ATCGTACGTGGGAGCCACTGC	61.5
miR-92a-2-5p	F: ATCGTACGTGGGAGGTGGGGA	62
miR-542-3p	F:ATCGTACGTGGGTGTGACAGA	57.5
miR-706	F:ATCGTACGTGGGAGAGAAACC	57.5
Common reverse primer	R: GCAGGGTCCGAGGTATTC	
GAPDH	F: CATGGCCTTCCGTGTTCCTA	62
GAPDH	R: CCTGCTTCACCACCTTCTTGAT	

F: forward, R: reverse.

A PrimeScript RT reagent Kit (Takara) was used to reverse-transcribe the RNA. The 10 μL reverse transcription reactions contained approximately 40 ng (6 μL) of small RNA, 2 μL of 5 × PrimeScript Buffer (Takara), 0.5 μL PrimeScript RT Enzyme Mix I (Takara), 1 μL of RNAase inhibitor (Takara), and 0.5 μL of a specific RT primer (10 μM). Temperature was maintained at 42°C for 30 min, 85°C for 5 s, and held at 4°C. Real-time PCR was performed in a 20 μL reaction mixture containing 2 μL of cDNA, 10 μL of 2 × SYBR Primer Ex TagII (TaKaRa, China), 6 μL of H_2_O, 1 μL of specific forward primers (Table 1, 10 μM), and 1 μL of a common reverse primer (10 μM). In parallel, a random primer (Takara) was used to synthesize cDNA for determining the abundance of the mRNA encoding glyceraldehyde 3-phosphate dehydrogenase (GAPDH, 10 μM) as the internal control. The reactions were amplified using a Master cycler ep realplex (Eppendorf, Germany) real-time PCR detection system, using the thermal cycling profile as follows: 95°C for 10 s, followed by 40 cycles of amplification at 95°C for 5 s, annealing for 30 s as indicated temperature, and 72°C 8 s. The value of each miRNA was normalized to that of GAPDH, because we demonstrated previously that GAPDH is stable in infected and uninfected mice [15]. The relative change between the control and infected samples was calculated using the 2^-ΔCt^ method [19].

### Prediction of target genes of altered levels of miRNAs: Gene ontology and KEGG pathway analyses

The miRWalk database (http://www.umm.uni-heidelberg.de/apps/zmf/mirwalk/) was queried to identify miRNA target genes. Only miRNA-target genes identified by at least four prediction programs were considered for further analysis. DAVID bioinformatics resources (http://david.abcc.ncifcrf.gov/) were used to identify gene ontology (GO) categories and were combined with Kyoto Encyclopedia of Genes and Genomes (KEGG) pathway analysis. The false discovery rate (FDR) was calculated to correct the *P* value. The lower the FDR, the lower the error in judging the *P* value. We defined enriched GO categories as those with *P* values < 0.05 and FDRs < 0.05. For KEGG pathway analysis, a *P* value < 0.05 and FDR < 0.1 were defined as significant.

### Analysis of the expression of selected miRNAs and their target genes

To investigate the link between altered levels of miRNAs and the expression of putative targets, qRT-PCR was performed to determine the expression of the genes encoding Caspase-3 for miR-706 [20], cAMP responsive element binding protein 1 (Creb1) for miR-134 [21], and Bcl2/adenovirus E1B interacting protein 3 (Bnip3) for miR-210 [22] in *S. japonicum* infected mice. Total RNAs were isolated from the plasma of mice at 25 dpi and transcribed into cDNA using a PrimeScript RT reagent Kit (Takara) and qRT-PCR analysis was performed using the SYBR Premix ExTaq kit (TaKaRa), each according to the manufacturer’s instructions. All PCR reactions were performed in a 20 μL reaction mixture under the conditions: 95°C for 30 s, 35 cycles at 95°C for 5 s, 60°C for 30 s, and 72°C for 8 s. The GAPDH gene was used as internal reference. Gene expression levels in each sample were calculated as described above. Primer sequences are listed in Table 2.

**Table 2:** **Primers for qRT-PCR analysis of miRNA target expression**

**Gene**	**GenBank ac.**	**Primer sequence 5'-3'**	**Product (bp)**
Bnip3	NM_009760.4	F: GCAATGGCAATGGGAGCA	146
		R: TGGTGTCTGGGAGCGAGGT	
Creb1	NM_001037726.1	F: AGCAGACAACCAGCAGAG	102
		R: GATACCTGGGCTAATGTGG	
Caspase-3	NM_009810.3	F: CTGACTGGAAAGCCGAAAC	204
		R: GGACTGGATGAACCACGAC	

F: forward, R: reverse, Bnip3: BcL2/adenovirus E1B interacting protein 3, Creb1: cAMP responsive element binding protein 1.

The levels in livers of these miRNAs and their putative targets were determined 25 dpi. Total RNAs from the livers of infected and uninfected mice was isolated using TRIzol (Invitrogen) with minor modification of the manufacturer’s instructions. Briefly, the RNA preparations were mixed with isopropyl alcohol and stored at −80°C for analysis of small RNA. Precipitated RNA was harvested using centrifugation and reserve-transcribed as described above. For miRNA stem loop qRT-PCR analysis, the 10 μL reverse transcription reactions contained 6 μL of small RNA enriched total RNA, 2 μL of 5 × PrimeScript Buffer (Takara), 0.5 μL of PrimeScript RT Enzyme Mix I (Takara), 1 μL of RNAase inhibitor (Takara), and 0.5 μL of a specific RT primer (10 μM). PCR reaction were performed using a SYBR Green PCR kit (Takara) as described above and contained 2 μL cDNA diluted 1: 10 in a final volume of 20 μL.

## Results

### Altered levels of circulating miRNAs in *S. japonicum*-infected mice

**Figure 1:**
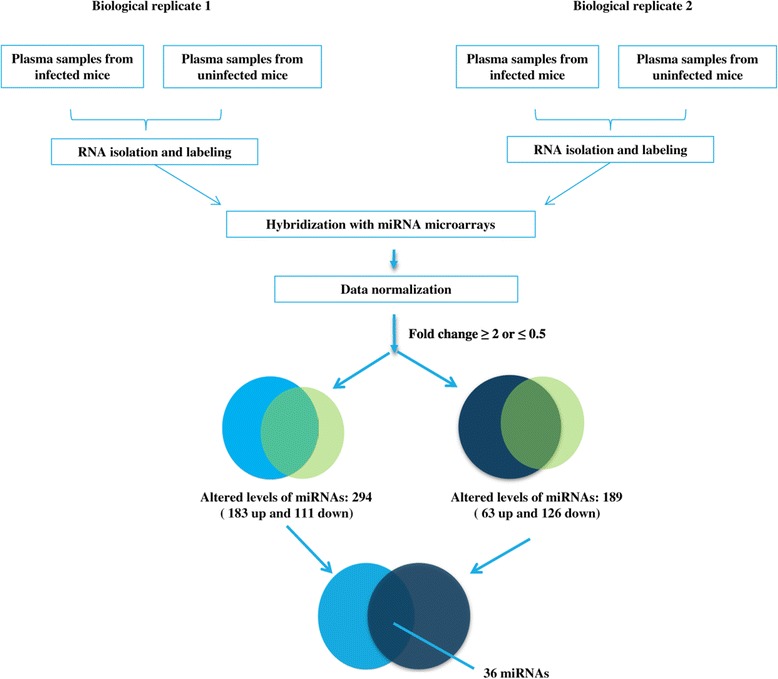
**Workflow of the analyses of altered levels of miRNAs in the plasma of schistosome-infected mice.**

**Figure 2:**
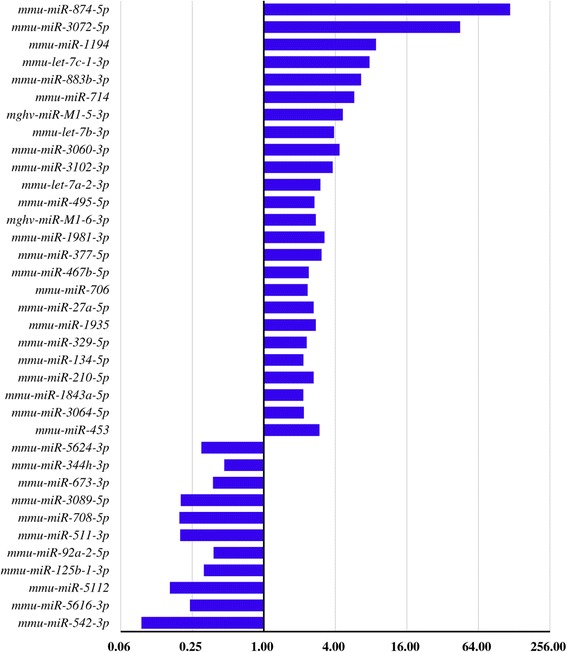
**Altered levels of miRNAs identified in two biological replicates (data represent the average difference in levels between the two biological replicates).**

We determined the levels of miRNAs in the plasma of mice infected with *S. japonicum* at 25 dpi compared with uninfected mice. The workflow of these analyses is summarized in Figure 1. We detected 294 and 189 miRNAs that were expressed at levels ≥ 2-fold higher and ≤ 0.5-fold lower compared with uninfected mice, respectively (Figure 1). Further, there were 36 miRNAs in the two biological replicates with consistent expression patterns (Figure 1). Of these, 11 miRNAs were down-regulated and 25 were up-regulated (Figure 2). Among them, mmu-miR-874-5p showed the greatest increase of the up-regulated miRNAs and those of mmu-miR-673-3p, mmu-miR-5112, mmu-miR-711, and mmu-miR-542-3p showed the greatest decrease of the down-regulated miRNAs (Figure 2).

### Validation of microarray results using qRT-PCR

**Figure 3:**
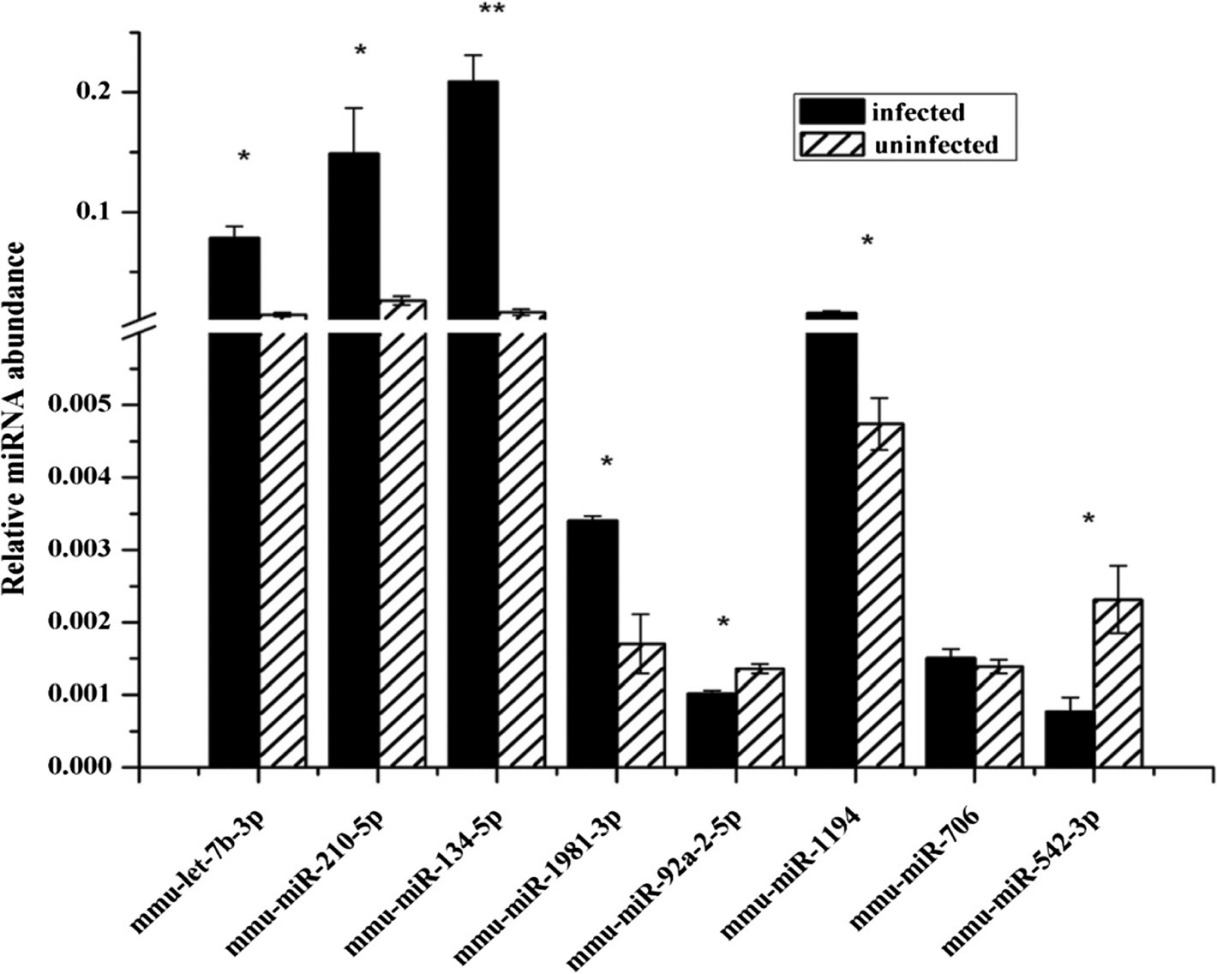
**Quantitative RT-PCR analyses of the levels of selected miRNAs in infected mice.** Data illustrate representative experiments and show the mean and standard error derived from triplicate biological replicates. Pools of plasma from at least four *S. japonicum* infected mice and four uninfected controls were used in each biological experiment. *means *P* ≤ 0.05 and **means *P* ≤ 0.01 (student’s t test analysis).

To validate the relative differences in the levels of miRNAs identified using the microarray, eight miRNAs were randomly selected for further qRT-PCR analysis. The plasma RNA was isolated from the pooled blood samples collected from mice infected with *S. japonicum* at 25 dpi. As shown in Figure 3, our results indicated that the levels of mmu-miR-134-5p, mmu-miR-1981-3p, mmu-let-7b-3p, mmu-miR-1194, and mmu-miR-210-5p were increased and the levels of mmu-miR-92a-2-5p and mmu-miR-542-3p were decreased in infected mice compared with those of uninfected mice. Although the level of mmu-miR-706 in the plasma of *S. japonicum* infected mice was increased, we did not observe obvious increase (Figure 3). Overall, these results suggest that the results of our microarray analysis are reliable.

### Biological function of the deregulated miRNAs

**Figure 4:**
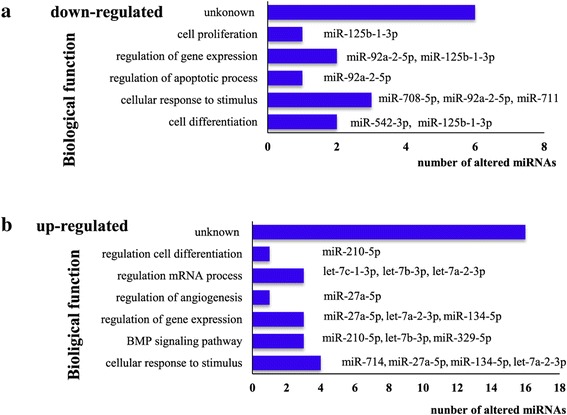
**Putative functional annotation of altered levels of miRNAs** **(down-regulated miRNAs for a and up-regulated miRNAs for** **b).** A full list of the biological functions of the differentially altered levels of miRNAs in 25 dpi mice compared with those detected in uninfected mice is shown in Additional file 1: Table S1.

The main functions of the 36 altered levels of circulating miRNAs (with reported function) associated with *S. japonicum* infection are summarized in Figure 4 (Additional file 1: Table S1). Most of the differentially regulated miRNAs are annotated as involved in cellular processes, with the direct function of cellular response to stimulus, differentiation, and apoptosis. Among them, seven miRNAs including three that were down-regulated (mmu-miR-708-5p, mmu-miR-92a-2-5p, and mmu-miR-711) and four that were up-regulated (mmu-miR-714, mmu-miR-134-5p, mmu-let-7a-2-3p, and mmu-miR-27a-5p) were predicted to be involved in cellular response to stimulus. Further, seven miRNAs were predicted to be related to the regulation of RNA expression, with the putative functions of mRNA process regulation (mmu-let-7a-2-3p, mmu-let-7b-3p, and mmu-let-7c-1-3p) and regulation of gene expression (mmu-miR-92a-2-5p, mmu-miR-125b-1-3p, mmu-let-7a-2-3p, mmu-miR-134-5p, and mmu-miR-27a-5p). Further, three other up-regulated miRNAs (mmu-miR-210-5p, mmu-miR-329-5p, and mmu-let-7b-3p) are proposed to be involved in the Bone morphogenic protein (BMP) signaling pathway.

### MiRNA target gene prediction, GO and KEGG pathway analyses

**Figure 5:**
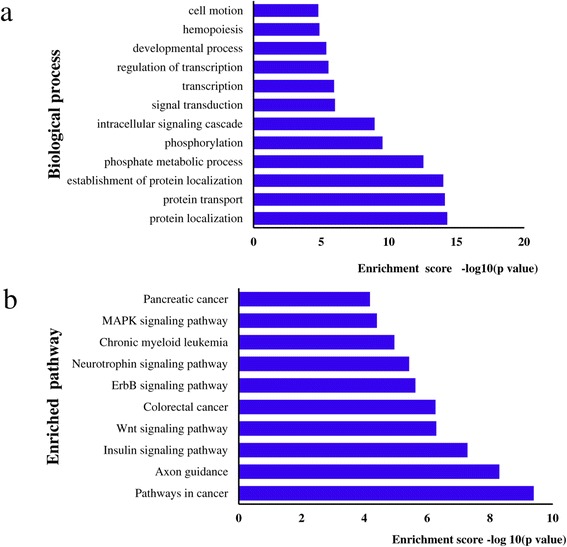
**Gene ontology categories and pathway analyses of the predicted target genes. a**. Categorization of miRNA-target genes was performed according to the biological process; **b**. Pathway analysis of the predicted target genes. The results of KEGG analysis of these altered miRNAs in 25 dpi *S. japonicum* infected mice compared with the uninfected mice are shown in Additional file 1: Table S2.

To gain insight into the functions of altered levels of miRNAs, the target genes of these miRNAs were identified using the miRwalk database. The predicted target genes were classified according to GO and KEGG functional annotations. As summarized in Figure 5, categories based on biological processes reveal that the predicted target genes are mainly involved in protein localization, transcription, intracellular signaling cascades, phosphate signaling processes, and phosphorylation (Figure 5a). KEGG pathway analyses indicates that 10 metabolic pathways are subject to regulation by the altered levels of miRNAs, such as Pathways in cancer, Axon guidance, Wnt signaling pathway, Neurotrophin signaling pathway, and MAPK signaling pathway (Figure 5b). Among them, Pathways in cancer was one of the most highly represented pathways affected by 121 target genes of the altered levels of miRNAs (Additional file 1: Table S2). Eighty-eight target genes were assigned to the MAPK signaling pathway (Additional file 1: Table S2), which is involved in a wide range of cellular responses, including gene expression, differentiation, proliferation and apoptosis [23], and the growth of malignant tumors [24].

### Analysis of the levels of selected miRNAs and their target genes

**Figure 6:**
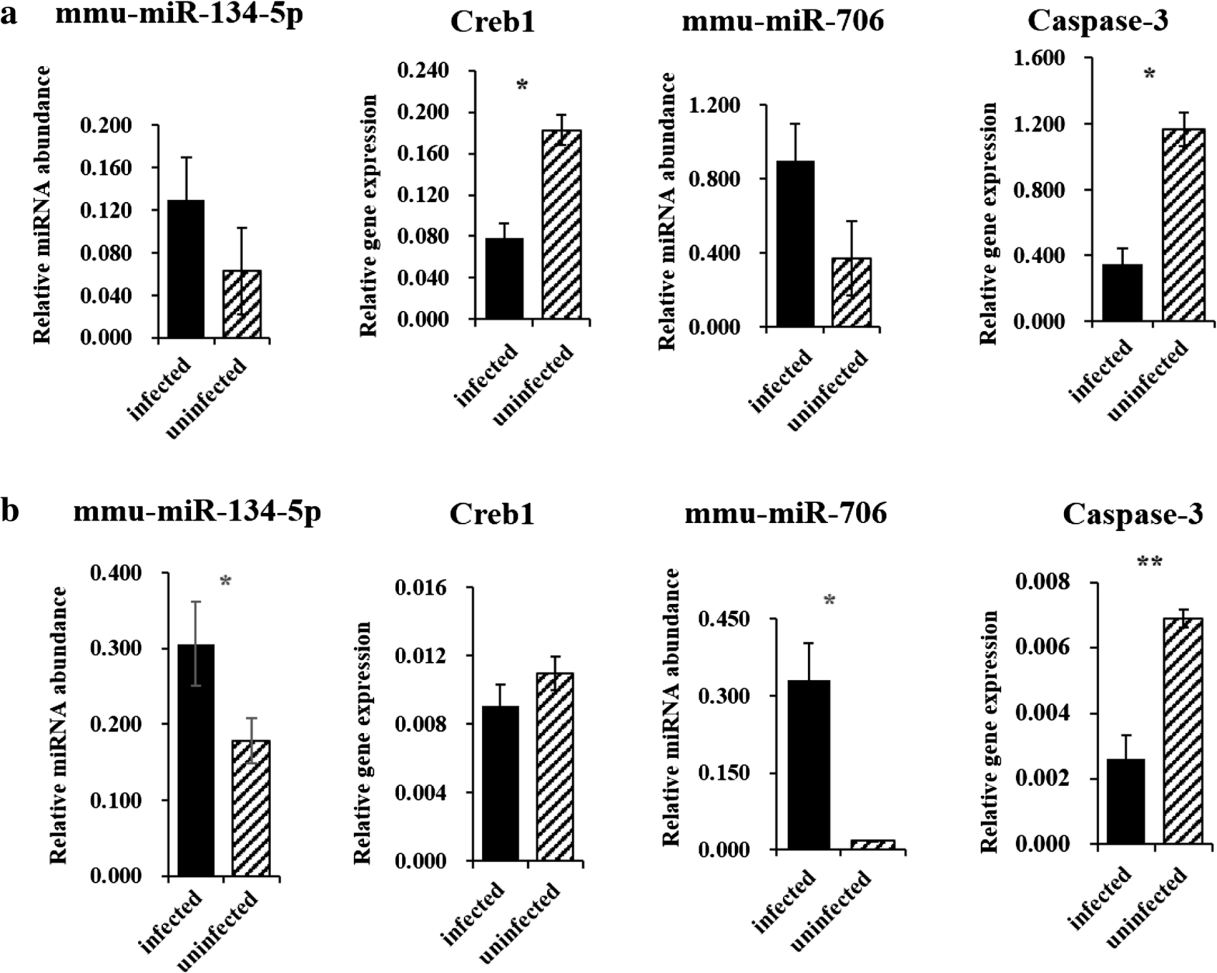
**Analysis of the levels of selected miRNAs and their target genes using qRT-PCR. a**. qRT-PCR analysis of the plasma levels of selected miRNAs and their target genes; **b**. qRT-PCR analysis of the levels of selected miRNAs and their target genes in the livers of *S. japonicum* infected mice. Data illustrate representative experiments and show the mean and standard error derived from triplicate biological replicates. Pools of plasma and livers from at least four *S. japonicum* infected mice and four uninfected controls were used in each biological experiment. Creb1: cAMP responsive element binding protein 1. *means *P* ≤ 0.05 and **means *P* ≤ 0.01. (student’s t test analysis).

We next determined whether there were changes in the levels of the target genes of the miRNAs that were differentially expressed in infected mice at 25 dpi. For this purpose, we selected three validated targets (Caspase-3, Creb1, and Bnip3) and then analyzed their expressions using qRT-PCR analysis. As shown in Figure 6a, there was an inverse correlation between the three selected miRNAs and the levels of their target mRNAs except for that of Bnip3, which was not significantly down regulated in the plasma of *S. japonicum* infected mice. Moreover, we showed that inverse correlation between the levels of these two miRNAs and their target mRNAs in infected mice liver (Figure 6b).

## Discussion

In the present study, we report, for the first time to our knowledge, the altered levels of circulating miRNAs associated with *S. japonicum* infection of mice. We found that 36 miRNAs were consistently altered in two independently biological replicates. Bioinformatic analyses indicate that these miRNAs are predicted to be involved primarily in cell differentiation, development, and cellular response. We show further that the expression of miRNA-target genes was inversely related to three altered levels of miRNAs in *S. japonicum* infected mice at 25 dpi. Because miRNAs in plasma are potential biomarkers for schistosomiasis diagnosis or as mediators of host-parasite interactions [8,15], the identification of different circulating miRNAs in schistosome infected hosts will expand our understanding of the mechanisms of the pathogenesis of this disease and may also contribute to the identification of novel biomarkers for diagnosing schistosomiasis.

To better define the functions of these miRNAs, KEGG analyses were performed to identify the signaling pathways of the predicted miRNA target genes. The results suggest that the MAPK and Wnt signaling pathways are most closely associated. Dysregulation of these pathways is associated with development of many types of cancer, in humans as well as in laboratory animals [25-27]. The altered levels of miRNAs might co-modulate these two pathways. Previous studies found an enrichment of MAPK and Wnt pathways in schistosoma infection [28,29]. For example, IL-10 production (an important anti-inflammatory cytokine) expressed at low levels in human schistosomiasis [30] requires the phosphorylation of p38 MAPK [31]. Moreover, a recent study indicates that MAPK phosphorylation and hypoxia-inducible factor-1alpha co-regulate the activation of hypoxic hepatic stellate cells that play a crucial role in the pathogenesis of liver fibrosis [32]. Consistent with these findings is the high-enrichment of the GO terms “regulation of phosphorylation” in the present study. These results support the hypothesis that the MAPK and Wnt signaling pathways are involved in the dysregulation of pathological reactions that occur during *S. japonicum* infection [28,29]*.*

Gene expression is typically down-regulated by miRNAs. We hypothesized that aberrant levels of miRNAs may lead to the altered expression of their target genes during schistosome infection. By searching validated miRNA target databases (miRwalk and miRBase database), we found that only eight miRNAs identified here have validated target gene; however, most miRNA target genes require validation in the future (Additional file 1: Table S3). Further analysis using qRT-PCR indicated that the levels of three selected target genes were inversely related to the levels of the corresponding miRNAs in plasma or liver of mice infected with *S. japonicum* (Figure 6), suggesting miR-706, miR-210-5p, and miR-134-5p regulate the expression of genes encoding their respective targets (Caspase-3, Bnip3, and Creb1, respectively) during *S. japonicum* infection of the final host. Among them, it is noteworthy that the level of mmu-miR-706 was inversely related to that of its target gene Caspase-3 in the plasma and livers of mice infected with *S. japonicum* at 25 dpi. Activated Caspase-3 mediates primarily the intrinsic apoptotic pathway by cleaving target proteins to execute cell death [33]. Therefore, our study suggests that apoptosis may be attenuated in the circulation and liver of a final host during *S. japonicum* infection [28,29]. In addition, we also investigated whether the changes of these target genes associated with altered levels of circulating miRNAs also occurred in the tissues isolated from lung, spleen, and kidney of *S. japonicum* infected mice at 25 dpi. However, we were not able to observe the inverse correlation in these organs of infected mice. It is most likely that the liver as one of important blood flow and supply organs may easily result in the enrichment of aberrantly expressed miRNAs into target cells and then these miRNAs significantly play a regulatory role in their corresponding target genes. Moreover, it is necessary to point out that one target gene can be simultaneously regulated by multiple miRNAs. Consequently, it remains to further determine whether other circulating miRNAs are also involved in the regulation of these target genes, resulting into their inverse expressions, in future studies.

Normal and pathological states are influenced by miRNAs [34]. Dysregulation of the expression of miRNAs that act as tumor suppressors or oncomirs may influence tumorigenesis. Here, we detected low levels of miR-542-3p in the plasma of *S. japonicum* infected mice. MiR-542-3p is a tumor suppressor and plays a crucial role in preventing malignant transformation. For example, down-regulation of miR-542-3p levels is closely associated with tumor progression via c-Src-related oncogenic pathways [35]. Up-regulation of miR-542-3p regulates the cell cycle by inducing both G1 and G2/M arrest [36], which inhibits breast cancer progression [37]. Moreover, we found that *S. japonicum* infection up-regulated miR-706 levels in the plasma of infected mice, and miR-706 and miR-542-3p co-regulate target genes according to DAVID analysis. Interestingly, miR-706 regulates cell differentiation, and up-regulation of miR-706 decreases vesicular stomatitis virus-induced apoptosis in BHK cells [20]. In the present study, our results show that down-regulation of caspase-3, a putative target of miR-706, combined with the high level of miR-706 and down-regulation of miR-542-3p expression, suggests cellular apoptosis may to some extent be inhibited in *S. japonicum* infected mice.

Other miRNAs play important roles in parasitic infections or in the cell cycle process. For example, miR-210 levels are increased in many tissues, and are directly and indirectly involved in the cell cycle, development, differentiation, amino-acid catabolism, and apoptosis [38]. Consistent with the results of previous studies [16], we show here that the levels of miR-210 were increased in the plasma of mice infected with *S. japonicum* 25 dpi, which is consistent with the analysis by Hoy et al. [16]. Further, let-7b regulates neural stem cell proliferation and differentiation [39,40]. Following acute stress, let-7b and let-7c are up-regulated in the frontal cortex of mice [41]. Here, we detected enrichment of let-7a-2-3p, let-7c-1-3p, and let-7b-3p in *S. japonicum* infected mice, suggesting that the homeostasis of *let-7* family miRNAs might play a critical role in regulating of cell differentiation in the *S. japonicum* infected mice; however, determining the roles of let-7 s in *S. japonicum*-infected mice requires further studies.

Cancer progression is associated with miR-92a and miR-134 that acts as oncomirs to promote cell proliferation, migration, and invasion [42,43]. Different types of cancer cells express either high or low levels of miR-92 [44-46] and the high levels of circulating miR-134 was proposed as a diagnostic and prognostic biomarker for certain diseases [47,48]. Combining the use of miR-134 and other miRNA biomarkers may enhance the diagnostic sensitivity of detection of mild cognitive impairment [48]. Therefore, the dysregulation of these miRNAs in the plasma of mice induced by schistosome infection may represent a mechanism associated with schistosomiasis progression. In addition, it must be keep in mind that, in the present study, we determined aberrant levels of circulating miRNAs in mice infected with *S. japonicum* at 25 dpi, which is an early stage of the liver pathology of schistosomiasis. It is possible that the different levels of circulating miRNAs in the stages associated with hepatic granulomatous and liver fibrosis may also play important roles in schistosomiasis progression, which need to be further investigated in future studies.

## Conclusions

In summary, our study shows significant differences in the levels of circulating miRNAs between *S. japonicum* infected mice and uninfected mice. Using complex pathway analyses, different KEGG pathways and GO terms were identified that associated with the altered expression of targets of miRNAs. In particular, the altered levels of miR-706 and miR-134-5p were associated with altered levels of expression of the Caspase-3 and Creb1 genes, respectively, which may serve as important mediators of the pathology of hepatic schistosomiasis. Our results will aid in the understanding of the potential roles of miRNAs in host-parasite interaction and the pathology of schistosomiasis.

## Additional file

Additional file 1: Table S1. Prediction and function annotation of the altered miRNAs. **Table S2.** Pathway analysis of the predicted target genes. **Table S3.** Validated target gene set of the altered miRNAs from miRwalk and miRBase database.
