# Supplementary material for: Altered levels of circulating miRNAs are associated Schistosoma japonicum infection in mice
Source: Parasit Vectors. 2015 Apr 1;8:196. doi: 10.1186/s13071-015-0806-5 (PMC4391475; doi:10.1186/s13071-015-0806-5)
Supplement: Additional file 1: Table S1. — Prediction and function annotation of the altered miRNAs. Table S2. Pathway analysis of the predicted target genes. Table S3. Validated target gene set of the altered miRNAs from miRwalk and miRBase database. [file 13071_2015_806_MOESM1_ESM.docx]

**Supplementary Tables**

Table S1. Prediction and function annotation of the altered miRNAs

| Name | Biological function | Entrez Gene Acc. | Chromosome |
| --- | --- | --- | --- |
| Down-regulation | | | |
| mmu-miR-542-3p | hematopoietic stem cell differentiation | 723901 | X |
| mmu-miR-708-5p | cellular response to amino acid stimulus | 735284 | 7 |
|  | cellular response to estrogen stimulus |  |  |
| mmu-miR-92a-2-5p | negative regulation of B cell apoptotic process | 723942 | X |
|  | negative regulation of apoptotic process |  |  |
|  | cellular response to amino acid stimulus |  |  |
|  | regulation of gene expression |  |  |
| mmu-miR-711 | response to oxygen levels | 751536 | 9 |
| mmu-miR-125b-1-3p | cellular response to estrogen stimulus | 387236 | 9 |
|  | cellular response to inorganic substance |  |  |
|  | common myeloid progenitor cell proliferation |  |  |
|  | hematopoietic stem cell differentiation |  |  |
|  | juxtaglomerular apparatus development |  |  |
|  | positive regulation of gene expression |  |  |
| Up-regulation | | | |
| mmu-miR-714 | cellular response to amino acid stimulus | 100124455 | Unknown |
| mmu-miR-210-5p | BMP signaling pathway | 387206 | 7 |
|  | negative regulation of activin receptor signaling pathway |  |  |
|  | positive regulation of osteoblast differentiation |  |  |
| mmu-miR-134-5p | cellular response to amino acid stimulus |  | 12 |
|  | negative regulation of gene expression |  |  |
| mmu-miR-329-5p | BMP signaling pathway | 723842 | 12 |
| mmu-miR-27a-5p | cellular response to glucose stimulus | 387220 | 8 |
|  | cellular response to lipopolysaccharide |  |  |
|  | positive regulation of angiogenesis |  |  |
|  | regulation of gene expression |  |  |
|  | regulation of semaphorin-plexin signaling pathway |  |  |
|  | retina vasculature development in camera-type eye |  |  |
| mmu-let-7a-2-3p | cellular response to amino acid stimulus | 723965 | 9 |
|  | cellular response to lipopolysaccharide |  |  |
|  | embryo implantation |  |  |
|  | negative regulation of gene expression |  |  |
|  | regulation of gene silencing by miRNA |  |  |
| mmu-let-7b-3p | BMP signaling pathway | 387245 | 15 |
|  | embryo implantation |  |  |
|  | mRNA catabolic process |  |  |
|  | response to oxygen levels |  |  |
| mmu-let-7c-1-3p | embryo implantation | 387246 | 16 |
|  | gene silencing by miRNA |  |  |
|  | mRNA catabolic process |  |  |

Table S2. Pathway analysis of the predicted target genes

| KEGG pathway | Targets | P value | FDR |
| --- | --- | --- | --- |
| mmu05200:Pathways in cancer | 121 | 4.04E-10 | 5.03E-07 |
| mmu04360:Axon guidance | 60 | 5.04E-09 | 6.27E-06 |
| mmu04910:Insulin signaling pathway | 60 | 5.26E-08 | 6.55E-05 |
| mmu04310:Wnt signaling pathway | 61 | 5.16E-07 | 6.43E-04 |
| mmu05210:Colorectal cancer | 41 | 5.44E-07 | 6.77E-04 |
| mmu04012:ErbB signaling pathway | 40 | 2.39E-06 | 2.98E-03 |
| mmu04722:Neurotrophin signaling pathway | 53 | 3.76E-06 | 4.69E-03 |
| mmu05220:Chronic myeloid leukemia | 35 | 1.11E-05 | 1.39E-02 |
| mmu04010:MAPK signaling pathway | 88 | 4.02E-05 | 5.01E-02 |
| mmu05212:Pancreatic cancer | 32 | 6.70E-05 | 8.34E-02 |

FDR：False discovery rate.

Table S3. Validated target gene set of the altered miRNAs from miRwalk and miRBase database

| miRNA | miR_Chr. | Validated target genes |
| --- | --- | --- |
| mmu-miR-883b-3p | X | Dicer1 Mbp Lin28 |
| mmu-miR-714 | 0 | Tlx2 |
| mmu-miR-706 | 6 | Caspse3 Akt1 Irs1 Caspse9 Slc3a2 |
| mmu-miR-1194 | 4 | Dicer1 |
| mmu-miR-453 | 12 | Esr1 |
| mmu-miR-673-3p | 12 | Mbp Lin28 |
| mmu-miR-542-3p | X | Ncam1 Acsm4 Sfn Lhx2 Neurog1 Ascl1 Omp Foxg1 Mbp Elavl4 Lin28 Rela Gnao1 Gnai2 Neurod1 Survivin |
| mmu-miR-134-5p | 12 | Creb1 |
